# International e-Delphi Consensus Recommendations for the Assessment and Diagnosis of Circadian rest–Activity Rhythm Disorders (CARDs) in Patients with Cancer

**DOI:** 10.3390/cancers15153784

**Published:** 2023-07-26

**Authors:** Craig Gouldthorpe, Sonia Ancoli-Israel, Elizabeth Cash, Pasquale Innominato, Gunnhild Jakobsen, Francis Lévi, Christine Miaskowski, Arti Parganiha, Atanu Kumar Pati, Deidre Pereira, Victoria Revell, Jamie M. Zeitzer, Andrew Davies

**Affiliations:** 1 School of Medicine, Trinity College Dublin, D02 PN40 Dublin, Ireland; 2 Academic Department of Palliative Medicine, Our Lady’s Hospice & Care Services, D6W RY72 Dublin, Ireland; 3 Department of Psychiatry, University of California San Diego School of Medicine, La Jolla, CA 92093, USA; 4 Department of Otolaryngology-Head & Neck Surgery & Communicative Disorders, UofL Healthcare-Brown Cancer Center, University of Louisville School of Medicine, Louisville, KY 40202, USA; 5 Oncology Department, Alaw, Ysbyty Gwynedd, Betsi Cadwaladr University Health Board, Bangor LL57 2PW, UK; 6 Cancer Research Centre, Warwick Medical School, University of Warwick, Coventry CV4 7AL, UK; 7 UPR “Chronotherapy, Cancers and Transplantation”, Faculty of Medicine, Paris-Saclay University, 94800 Villejuif, France; 8 Department of Public Health and Nursing, Faculty of Medicine and Health Sciences, Norwegian University of Science and Technology (NTNU), 7491 Trondheim, Norway; 9 Cancer Clinic, St. Olavs Hospital, Trondheim University Hospital, 7006 Trondheim, Norway; 10 Gastro-Intestinal and General Oncology Service, Paul Brousse Hospital, Assistance Publique-Hôpitaux de Paris, 94800 Villejuif, France; 11 Division of Biomedical Sciences, Cancer Chronotherapy Team, Warwick Medical School, University of Warwick, Coventry CV4 7AL, UK; 12 Department of Physiological Nursing, University of California, San Francisco, CA 94143, USA; 13 School of Studies in Life Science & Centre for Translational Chronobiology, Pandit Ravishankar Shukla University, Raipur 492010, India; 14 Odisha State Higher Education Council, Government of Odisha, Bhubaneswar 751001, India; 15 Kalinga Institute of Social Sciences, Bhubaneswar 751024, India; 16 Department of Clinical and Health Psychology, University of Florida Health, Gainesville, FL 32610, USA; 17 Surrey Sleep Research Centre, University of Surrey, Surrey GU2 7XH, UK; 18 Department of Psychiatry and Behavioral Sciences, Stanford University, Stanford, CA 94305, USA; 19 Mental Illness Research Education and Clinical Center, VA Palo Alto Health Care System, Palo Alto, CA 94304, USA; 20 School of Medicine, University College Dublin, D04 V1W8 Dublin, Ireland

**Keywords:** cancer, circadian rhythm, diagnosis, symptoms, quality of life, survivorship, delphi

## Abstract

**Simple Summary:**

Circadian rhythms are internal changes that happen roughly over a 24-h period. In patients with cancer, healthy circadian rhythms may become abnormal, and these changes have been linked to more symptoms, poorer quality of life, and shorter survival. Circadian rhythms can be observed in rest and during physical activity, and disorders of this rest–activity rhythm are common. There are many ways to research and report rest–activity rhythms, and currently, there are no formal criteria to diagnose the condition. This study aimed to provide recommendations on how to assess and diagnose the condition.

**Abstract:**

Purpose: Circadian rest–Activity Rhythm Disorders (CARDs) are common in patients with cancer, particularly in advanced disease. CARDs are associated with increased symptom burden, poorer quality of life, and shorter survival. Research and reporting practices lack standardization, and formal diagnostic criteria do not exist. This electronic Delphi (e-Delphi) study aimed to formulate international recommendations for the assessment and diagnosis of CARDs in patients with cancer. Methods: An international e-Delphi was performed using an online platform (Welphi). Round 1 developed statements regarding circadian rest–activity rhythms, diagnostic criteria, and assessment techniques. Rounds 2 and 3 involved participants rating their level of agreement with the statements and providing comments until consensus (defined internally as 67%) and stability between rounds were achieved. Recommendations were then created and distributed to participants for comments before being finalized. Results: Sixteen participants from nine different clinical specialties and seven different countries, with 5–35 years of relevant research experience, were recruited, and thirteen participants completed all three rounds. Of the 164 generated statements, 66% achieved consensus, and responses were stable between the final two rounds. Conclusions: The e-Delphi resulted in international recommendations for assessing and diagnosing CARDs in patients with cancer. These recommendations should ensure standardized research and reporting practices in future studies.

## 1. Introduction

Circadian rhythms describe endogenous bodily processes that are aligned over an approximate 24-h period and are coordinated by a central master clock within the suprachiasmatic nuclei of the hypothalamus [1]. External cues, or zeitgebers, align internal circadian rhythms with the environment [1]. To assess if circadian rhythms and circadian rhythm disorders possess functional or dysfunctional underlying clock machinery, they are investigated under controlled conditions where environmental cues are removed. In this article, circadian rhythms refer broadly to 24-h rhythmicity in real-world conditions and may also be termed diurnal rhythms.

One of the strongest zeitgebers, the presence or absence of light, is detected by the retina and communicated to the SCN, which, in turn, influences melatonin secretion and the sleep–wake cycle [1]. Other important zeitgebers include physical exercise and food intake [1]. Internal alignment is perpetuated through physiological circadian rhythms, including core body temperature and hormones [1].

Circadian rhythms may be strengthened by regularising our daily habits and the timing of exposure to zeitgebers. Altered timing, such as light exposure late in the evening, inhibits melatonin secretion and leads to a delay in our expected sleep timing [2]. Similarly, while considering an individual’s preference for the time of sleep (chronotype), the timing of exercise can delay or advance (bring forward) the sleep phase [3]. Acting more peripherally, the timing of food intake can also influence circadian rhythms [4]. Disruptions in circadian rhythms are common in patients with cancer and include disordered cortisol, melatonin, and rest–activity rhythms (RARs) [5,6,7]. These disruptions may be driven by circadian processes or alterations in physiology downstream from circadian clocks.

A clinically relevant circadian rhythm includes RARs, which reflect our daily patterns of physical activity and rest. RARs are related to, but distinct from, sleep–wake rhythms. RARs can differ among individuals in relation to the regularity of the RAR between days (interdaily stability), how distinct rest and active periods are distributed across the day (intradaily variability), and/or the difference between the highest and lowest activity levels over 24-h (relative amplitude). 

RAR characteristics differ by sex, age, and race. For example, compared to men, women demonstrate higher average and peak activity levels, less intradaily variability, and more interdaily stability of their RARs [8]. As we age, average and peak activity levels tend to reduce; peak activity occurs earlier in the day, and the RAR becomes more stable between days and less variable within the day [8]. Compared to White individuals, Black individuals tend to demonstrate less interdaily stability, while Asian individuals have a later timing of peak activity (acrophase) [8]. Accelerometry uses wearable devices to assess RARs, including in patients with cancers and allows for prolonged, non-invasive measurement of physical activity and sleep in various settings [9]. Data generated from accelerometry can be interpreted in several ways (Supplementary Materials Table S1), with variations in the research and reporting practices of RARs and their parameters [10].

Significant associations have been noted between RARs and health and disease across different populations. For example, a recent study involving 7252 American adults highlighted that stronger RARs, demonstrated by an increased relative difference between the most and least active time spans, were associated with reduced cancer-related, cardiovascular disease-related, and all-cause mortality [11]. Disrupted RARs occur in up to 54.9% of patients with advanced cancer, and those with disordered RARs have higher morbidity, including symptoms of fatigue, pain, anorexia, dyspnoea, depression, and anxiety, along with poorer general health-related quality of life and physical, social, and role function domains [7,10,12,13,14]. Objectively measured elevated night-time activity, using accelerometry, is also associated with subjective sleep difficulties, which is common among patients with cancer [15].

Several parameters, such as the dichotomy index (I < O), 24-h autocorrelation coefficient (r24) and sleep efficiency, are associated with survival across cancer groups, independent from other known prognostic factors. Although the underlying causative mechanisms remain unclear, a study of women with breast cancer found that levels of tumor invasion and immunosuppression biomarkers that play a role in cancer progression were lower in those with more robust RARs [16]. Survival examples include a 9-month shorter median overall survival and a 3-month shorter median progression-free survival in patients with metastatic colorectal cancer who have proportionally higher in-bed versus out-of-bed activity [17]. RAR disruption is associated with a shorter, 2-year overall survival for patients with head and neck cancers (HR 0.073, *p* = 0.012) [14]. Considering links between survival and specific accelerometry parameters, the dichotomy index (HR 0.95, *p* < 0.0001) and the 24-h auto-correlation coefficient (HR 0.20, *p* < 0.004) appear to be of importance [18]. In a recent meta-analysis, an altered dichotomy index was shown to be associated with a more than two-fold increased risk of earlier death in patients with cancer [19]. Disease-free survival is also shortened among patients where preferred and actual bedtimes are misaligned [20].

Currently, research and reporting practice in the assessment of RARs in patients with cancer lacks standardization. In turn, this limits the interpretation and application of research findings. Although parameters generated from accelerometry data may indicate RAR disturbance, no formal diagnostic criteria exist. The aim of this study was to create expert international recommendations on the assessment and diagnosis of Circadian rest–Activity Rhythm Disorders (CARDs) in patients with cancer.

## 2. Methodology

**Figure 1:**
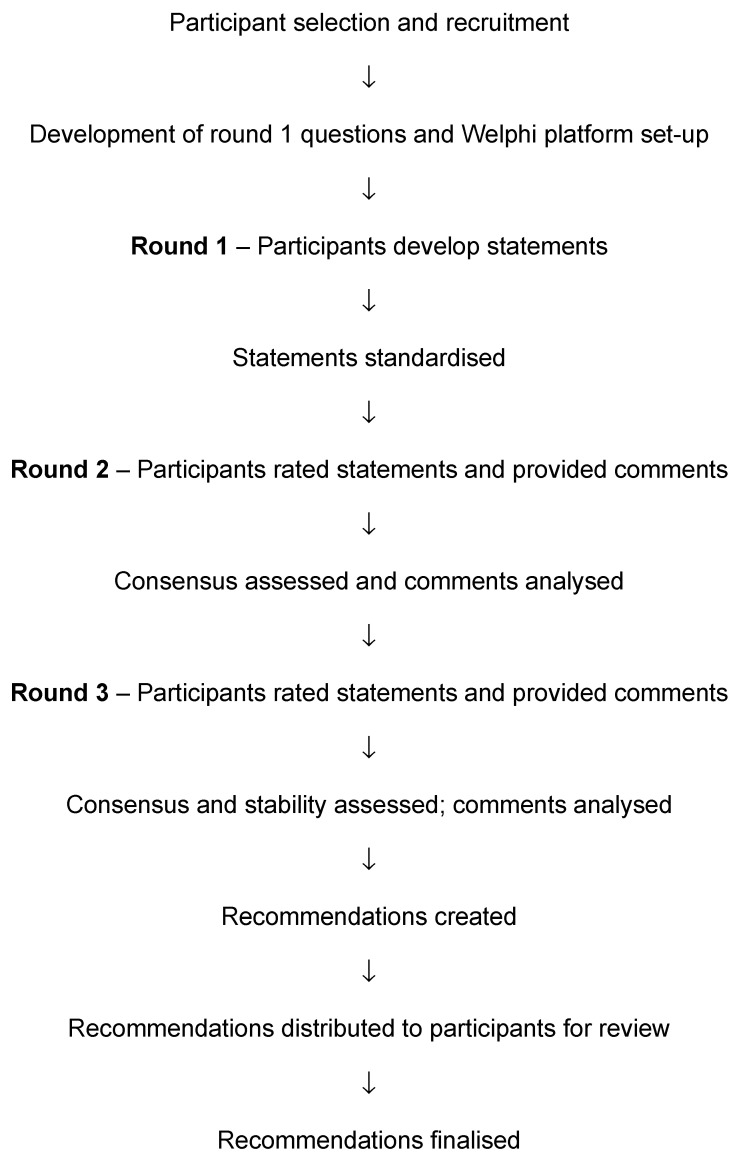
Flow chart of the e-Delphi process.

An electronic Delphi (e-Delphi) process, consisting of three rounds (see Figure 1), was carried out using the Welphi platform between August and December 2022. The project was sponsored by Our Lady’s Hospice and Care Services, received ethical approval from the St James’s Hospital–Tallaght University Hospital Joint Research Ethics Committee (project number 1926), and was registered on the Comet Initiative database (http://www.comet-initiative.org/studies/searchresults?guid=dd7711a4-4991-4931-972c-c539a641ce1a). The results of the e-Delphi process are reported in accordance with CREDES guidance [21].

Potential participants (“experts”) were defined as individuals with English proficiency who were actively participating in circadian activity rhythm research in cancer patients (“rest–activity” and/or “sleep–wake”). They were identified through the literature review (purposive sampling) and were invited to recommend additional participants who may be relevant to the e-Delphi aims (snowballing sampling). Identified participants were provided with information on the study processes and aims and asked to complete a study-specific consent form. No additional information on the research topic was provided. Participant responses remained anonymous throughout the process.

The e-Delphi process involved three rounds, with each round being open for three weeks. In Round 1, participants were asked to provide demographic data (e.g., location, job title, and years of engaging in rest–activity rhythm research in cancer patients) and asked to answer open and closed questions (Supplementary Materials Table S2) relating to rest–activity rhythms, diagnostic criteria, and assessment methods. Questions were reviewed by three researchers (CG, AD, JP) prior to the Welphi platform going live. Once complete, two researchers (CG, JP) reviewed the answers and generated standardized statements for Rounds 2 and 3.

Round 2 of the e-Delphi involved participants rating their level of agreement with these statements using a 5-point Likert scale (i.e., “Strongly Disagree”, “Disagree”, “Unsure”, “Agree”, “Strongly agree”) and providing relevant comments about these statements. “Unsure” was included as an option due to the novel nature of the topic. Participants were provided with personalized emails at the start and end of each round, along with reminder emails prior to round closure.

Round 3 of the e-Delphi was similar to Round 2, but participants were presented with overall group responses for each statement from Round 2, together with any anonymized comments that were made during Round 2. Round 3 included two new statements and one modified statement, which were suggested during Round 2. The aim was to achieve consensus (defined as at least 67% agreement) for the statements (i.e., majority agree, majority disagree, or majority unsure). We anticipated that due to this novel research area, and the inclusion of experts from different backgrounds, consensus may not be possible for all of the statements. Lack of consensus was also considered an important finding.

In addition to assessing the level of consensus, the stability of responses between rounds was assessed. Once stable consensus was achieved for several statements, the results were reviewed by two researchers (CG, AD) to develop recommendations on the assessment and diagnosis of CARDs in patients with cancer. These recommendations were then fed back to the participants for comments before being finalized.

### Interpretation and Processing of Results

Recruitment and retention rates were noted for each round using crude figures and percentages. Group ratings for each statement were reported for each round and reviewed to assess if consensus was achieved. Comments were reviewed independently by two researchers (CG, JP) using content analysis to assess if any modified or new statements needed to be created. When more than one round had taken place, Spearman’s rank correlation coefficients were used to assess stability between rounds for each statement, with values of 0.80–1.00 suggesting very strong associations and 0.60–0.79 suggesting strong associations between rounds. For a significance level of 0.05 and 13 participants, the critical value was 0.484.

## 3. Results

**Figure 2:**
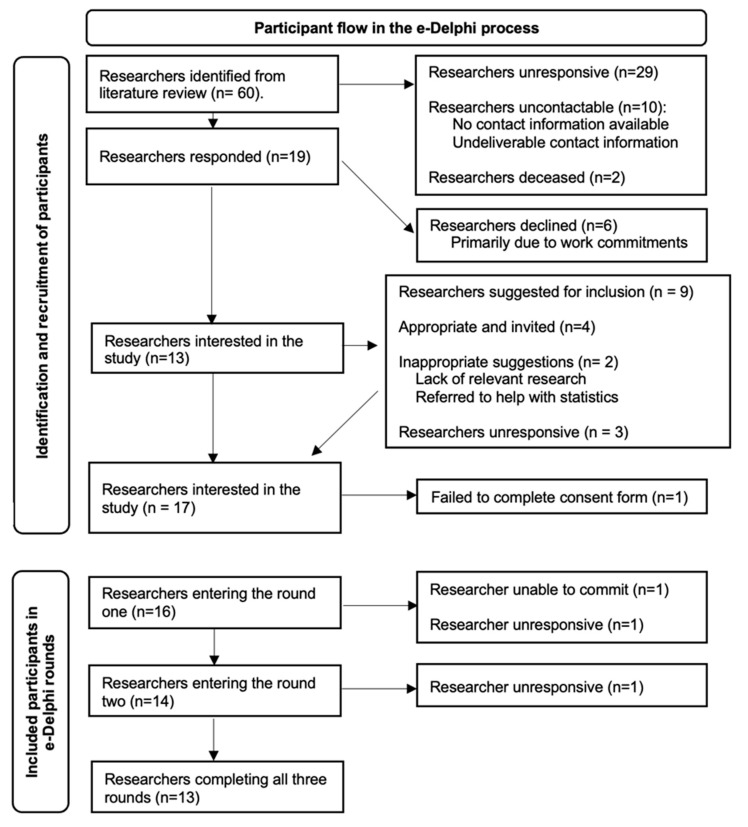
Participant flow in the e-Delphi process.

Sixty researchers were identified from the review of the literature, and a further nine researchers were suggested by the initially invited researchers. Sixteen researchers took part in the e-Delphi process, and thirteen researchers completed all e-Delphi rounds (see Figure 2). Participants were from nine different clinical specialties, working in seven different countries with 5–35 years of experience researching circadian activity rhythms in cancer patients (see Table 1). Participant retention and reasons for attrition between rounds are presented in Table 2.

Participants created 155 statements in Round 1, which were combined with 6 statements from an earlier literature review, creating 161 statements in total. Statements focused on circadian rest–activity rhythms, circadian rest–activity rhythm disorder diagnostic criteria, and assessment methodology (i.e., clinical history and physical examination, and further investigations). Following Round 2, 43 comments were made, which provided explanations of participant ratings (n = 25), suggested modifications (n = 13), suggested new statements (n = 3), or a combined explanation and suggested modification (n = 1). Two new statements and one modified statement were added. Group responses to each statement across rounds, along with stability between rounds, are reported in Supplementary Materials Table S3a–e.

After Round 3, consensus was achieved for 66% of the original statements and for one modified and one new statement. Of the remaining 56 statements, 22 had opposing disagree–agree ratings; 16 had an equal mix of ratings; 15 demonstrated skewness towards agree or disagree, and 3 demonstrated a majority for unsure. Statement responses between Rounds 2 and 3 demonstrated very strong agreement (Spearman’s rank correlation coefficient 0.80–1.0) for 80% of the statements and strong agreement (Spearman’s rank correlation coefficient 0.60–0.79) for 17% of the statements. With consensus agreement for many statements and agreement between rounds, the e-Delphi was terminated, and diagnostic criteria and recommendations were formulated (Table 3 and Table 4).

### 3.1. Definition of a Circadian Rest–Activity Rhythm Disorder in Patients with Cancer

Patients with cancer may develop a circadian rest–activity rhythm disorder (CARD), which involves an ongoing change in physical activity over a 24-h period (day and night), or between 24-h periods and is associated with increased morbidity and/or decreased quality of life.

Participants agreed that CARDs occur in patients with cancer and in neurological, respiratory, and psychiatric diseases, primary sleep disorders, and, more generally, in acute illness. Participants agreed that it is unknown whether CARDs differ significantly between diagnoses. Furthermore, the group agreed that there were modifiers of the rest–activity rhythms, including uncontrolled symptoms, chemotherapy, alcohol, sedatives, stimulants, shift work, and jet lag.

### 3.2. Lack of Consensus and Opposing Views

Responses to several statements demonstrated a bimodal distribution, whereby the majority of responses were split between disagree and agree. These results may represent two cohorts of experts. The statements included the minimum and maximum duration of accelerometry monitoring. The highest levels of agreement were 3 days for the minimum period and 14 days for the maximum period. Most participants disagreed that monitoring should take place for 30 days or more. Lack of consensus also related to the need for additional measures (bedroom activities, body temperature, and biomarkers such as melatonin), clinical history (family sleep patterns, fatigue not alleviated by rest), physical examination (necessity of a physical examination and measuring body mass index), and diary entries (pre-bedtime activities, time spent indoors and outdoors). Additionally, no consensus was formed on the duration of diary use, the duration of the disorder, accelerometry measures (least active 5 h, sleep onset latency, wake after sleep onset), and if actigraphy supports the diagnosis rather than being essential.

## 4. Discussion

**Figure 3:**
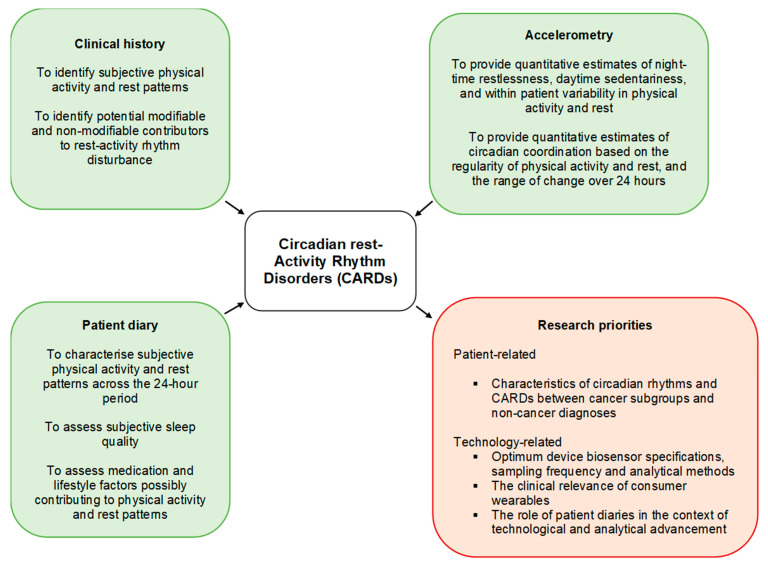
An overview of CARD assessment methods and future research priorities.

This e-Delphi has, for the first time, generated international, expert-derived, consensus recommendations for the assessment and diagnosis of circadian rest–activity rhythm disorders (CARDs) in patients with cancer. It is envisaged that embracing these recommendations will help to standardize research and reporting practices for this condition. An overview of CARD assessment methods, along with their relevance and future research priorities, has been outlined in Figure 3.

Recommended diagnostic criteria include a lack of regularity in RARs between days, poorly defined rest and active periods, and proportionally higher in-bed to out-of-bed activity. These recommendations are supported by objective measures, such as the dichotomy index (I < O) and 24-h autocorrelation coefficient (r24), which are associated with symptoms, quality of life measures, and survival among individuals with cancer [14,18,22].

Although the e-Delphi focused on RARs in patients with cancer, the recommendations highlight that disordered RARs can also occur in other clinical conditions. These include patients with neurodegenerative conditions (e.g., Parkinson’s disease and Alzheimer’s disease), psychiatric disorders (e.g., bipolar disorder), and respiratory disease (e.g., chronic obstructive pulmonary disease) [23,24,25,26]. Currently, evidence is insufficient to determine how RARs differ between diagnoses. Therefore, these recommendations cannot be extrapolated to patients with non-cancer conditions.

Several factors highlighted in the recommendations modify circadian RARs to varying extents and should be considered during an assessment. Complexity arises as disordered circadian activity in patients with cancer may predate anticancer therapies and, when used, anticancer therapy further contributes to a loss of the day–night distinction and worsening of RAR parameters, with disturbance seen for up to 4 months afterward [27,28,29]. Environmental modifiers are also important and include jetlag, which may impact psychometric measures, such as perceived jetlag and stress (up to 7 days), and physiological measures, such as grip strength, temperature, melatonin, and cortisol (up to 11 days) [30].

Consensus was not achieved for the minimum duration of accelerometry monitoring. In this case, the majority opinion, 72-h continuous monitoring, was accepted. Assessment of circadian RARs requires activity monitoring across 24-h periods, and, therefore, continuous monitoring is necessary. Recommendations for circadian sleep–wake rhythm disorders suggest assessments for 7 to 14 days, aiming to capture weekday and weekend activity [31]. As patients with advanced cancer exhibit similar activity levels and RARs on midweek and weekend days, prolonged monitoring may be unnecessary [32,33]. This recommendation is supported by existing studies commonly monitoring patients with cancer over 72-h, or 3 days, although a range of 72 h to 270 days has been used [34,35]. Longer periods of monitoring may be required, specifically if related to monitoring the impact of cancer or anticancer therapies on circadian rhythms. Studies have shown good patient adherence with accelerometry (73–97%) across the cancer trajectory, including cancer survivors and patients with advanced disease [34]. Various actigraphy devices are available, and the e-Delphi participants highlighted a need for ongoing standardization of device biosensor features, sampling frequency, and analytical methods. This was not assessed in this e-Delphi. In addition, this study focused on medical devices. However, consumer wearables are becoming more ubiquitous in the general population, including patients with cancer and cancer survivors, and evidence of their clinical relevance in decision-making and surveillance is increasing [36].

Various device locations have been used within cancer studies, including the wrist (as recommended), upper chest, hip, thigh, and ankle [34,35,37]. Recommendations highlight that device location should be confirmed when reporting accelerometry studies. The accuracy of the device location depends on the validation procedures and placement recommendations of the device manufacturer and the measure being considered, with the wrist site correlating well with moderate-to-vigorous activity but less so with sedentary or static activity [9,38]. This limitation, in providing a complete picture of physical activity, needs to be balanced against the burden of using multiple devices.

The recommendations include the use of patients’ diaries. Accurate diary entries require a good understanding of various physical activities and activity intensity [38]. To overcome recall bias, they require engaged individuals to complete entries as close to real time as possible [39]. Depending on the context, the individual’s perception of how desirable particular entries are may influence reporting [9]. Within sleep studies, diaries often overestimate sleep duration, sleep efficiency, and time in bed [40]. However, diaries may support more objective measures of activity and can contextualize their findings.

The heterogeneous sample of experts in relation to geographical location, research interest (rest–activity rhythms and/or sleep–wake rhythms), and research experience, while including academics and clinicians from several specialties, allowed for a broad perspective on the topic area. Thus, the recommendations represent a transdisciplinary approach to defining CARDs and ensure more generalizable recommendations [41].

Delphi methodology is assumed to be superior to individual opinion and draws on participants’ expertise and their understanding of the current evidence base [42]. Aside from detailing the purpose of the Delphi process in round 1, participants were not provided with additional information on the topic area, and several open-ended questions were used to generate statements for further rounds. This approach limited any bias from the research team and allowed for a wealth of information to be gathered prior to forming statements. The e-Delphi remained an online process with anonymity throughout and without direct interaction between participants. Anonymity aimed to reduce bias from dominant participants and group responses [41]. Following round 1, expert opinion was supported by evidence from the literature review of the area, and participant comments allowed for relevant research to be highlighted. The e-Delphi allowed for international engagement and for participants to balance engagement alongside other academic and clinical commitments. Alternative approaches to gain consensus opinion, such as the nominal group technique, would have required a more homogenous group and resulted in additional participant and researcher burden.

The majority (81%) of participants entering the e-Delphi completed all three rounds, which is comparable to other Delphi studies of outcome measures (majority at least 80%, range 45–100%) [43]. Previous Delphi studies reported completion rates in relation to those who initially entered the study or who completed the previous round. Both have been reported here for transparency. Similar to this study, other Delphi studies have consisted of three to four rounds lasting 10 days to 10 weeks [21,43]. Although five rounds may be acceptable, two rounds are recommended to reduce participant burden [42].

Prior to study commencement, consensus was determined internally as achieving at least 67% agreement. Consensus rates within other Delphi studies have ranged from 20–100%, with many using a threshold of at least 60% [42]. Within palliative care Delphi studies, higher thresholds of 75–80% have been used [21]. Of note, 89% of our statements reached the 75% threshold. Due to our small sample size, Spearman’s rank correlation coefficient was an appropriate measure of stability [44]. With consensus for several statements, stability between rounds, and strong engagement in feedback from participants, recommendations were formulated and provided to the participants for final comments. The validity of the recommendations was strengthened by providing the opportunity for further feedback before the recommendations were finalized. Additional rounds may have led to consensus on several other statements but with the risk of higher attrition. Similarly, focus groups or interviews may have provided additional information with the risk of additional participant and researcher burden, as well as the potential loss of anonymity and freedom from bias or influence.

## 5. Conclusions and Future Directions

The e-Delphi process generated international recommendations for the assessment and diagnosis of CARDs in patients with cancer. Further studies are needed to validate many of these recommendations, and ongoing investigations may result in adjustments to the recommendations. Future studies are needed to characterize RARs in different populations, including cancer subgroups at different stages of their disease and treatment trajectories, including long-term survivors, as well as non-cancer groups. Further research is required to identify affected patients earlier, assess management strategies to improve patients’ RARs, and, if successful, determine what impact this improvement has on clinical outcomes.

## Figures and Tables

**Table 1:** Recruited participant demographics (n = 16).

**Job title**	Professor (n = 7)
	Associate Professor (n = 4)
	Medical Consultant/Attending Physician (n = 3)
	Senior Lecturer (n = 1)
	No response (n = 1)
**Specialty**	Oncology (n = 3)
	Biosciences (n = 2)
	Psychiatry (n = 2)
	Psychology (n = 2)
	Sleep and Physiology (n = 2)
	Anaesthesiology (n = 1)
	Nursing (n = 1)
	Oncology, Palliative Medicine and Sleep (n = 1)
	Palliative Medicine (n = 1)
	No response (n = 1)
**Location**	United States (n = 5)
	UK (n = 3)
	India (n = 2)
	Norway (n = 2)
	Canada (n = 1)
	France (n = 1)
	Ireland (n = 1)
	No response (n = 1)
**Time spent researching activity rhythms in patients with cancer**	Median 15 years (range 5–35 years)

**Table 2:** Retention rates across e-Delphi rounds.

Retention Rates			
	Round 1	Round 2	Round 3
Of recruited participants	14/16	13/16	13/16
	87.5%	81.3%	81.3%
Of participants entering the round	14/16	13/14	13/13
	87.5%	92.9%	100%
Attrition reason	Unable to commit (1), no response (1)	IT problems / no response (1)	n/a

**Table 3:** Diagnostic criteria for circadian rest–activity rhythm disorders (CARDs) in patients with cancer.

The Patient Must Demonstrate an Altered Circadian Rest–Activity Rhythm Evidenced by One of the Following	Level of Agreement
Relatively less daytime and more night-time physical activity	92%
Rest and physical activity spread across the 24-h period, rather than in distinct rest and active periods	77%
A lack of regularity in rest and active periods between days.	77%
**The circadian rest–activity rhythm alteration must have all the following**	
Have been present for at least 1 month.	70%
Have a clinical impact on the patient.	84%
Be demonstrable by objective measures.	92%
Be demonstrable by subjective measures	69%
Not primarily be due to another cause.	NA

NA (not applicable): Statement agreed from e-Delphi findings, not assessed separately for level of agreement within the e-Delphi.

**Table 4:** Assessment approaches for circadian rest–activity rhythm disorders (CARDs) in patients with cancer.

A clinical history (“needed”; 92%), accelerometry (“essential”; 92%) and patient diary (“suggested”; 70%) are recommended to assess a CARD in people with cancer.	
**A clinical history should consider**	Level of agreement
An oncological history (cancer site, stage, site of metastases, and cancer treatments)	92%
The presence and timing of symptoms (e.g., fatigue, daytime sleepiness, pain)	100%
Medical, surgical, and psychiatric comorbidities	100%
Daily routine, type, and duration of physical activity	100%
If daytime sedentariness and/or night-time restlessness is present, the duration should be considered	100%
Sleep history, assessment of chronotype, and peak alertness	85–100%
Medication history (e.g., melatonin, beta blockers, steroids, stimulants, and sedatives)	100%
The use of tobacco, alcohol, caffeine, and illicit drugs	100%
Environmental factors (e.g., family, newborns, occupation, shift work, jet lag, noise and light exposure)	100%
**Assessment using accelerometry**	
Accelerometry should take place for at least 72 consecutive hours	LC
The location of an accelerometer device should be documented	NA
When using wrist actigraphy, the non-dominant wrist should be used unless contraindicated	100%
Removal of the device should be documented	93%
**Relevant accelerometry parameters**	
Evidence of night-time restlessness (e.g., sleep efficiency, number and duration of night-time awakenings)	77–84%
Evidence of daytime sedentariness (e.g., daytime sedentariness, number and duration of daytime naps)	85–92%
Evidence of daytime sedentariness and night-time restlessness (e.g., dichotomy index, physical activity relative amplitude, intra-daily variability)	85–92%
Evidence of a lack of regularity in rest and active periods between days (e.g., the 24-h autocorrelation coefficient, interdaily stability)	77–100%
Phase markers (e.g., most active 10 h (M10), activity acrophase	69–70%
**A patient sleep and activity diary**	
A sleep and activity diary supports accelerometry and the diagnosis of circadian rest–activity rhythm disorders	85–93%
A diary should consider day and night-time events and be used for the duration of accelerometry monitoring	NA
**Relevant information to document in a sleep and activity diary include**	
Time and duration of daytime naps	93%
Subjective daytime sleepiness	85%
Time, description, duration, and perceived level of exertion of physical activity	70–92%
Presence of symptoms during physical activity (e.g., pain or fatigue)	69%
Medication use	84%
Alcohol, smoking, caffeine, and substance use	85%
Bedtime	84%
Time to lights out	84%
Sleep onset	76%
Time and duration of night-time awakenings	85%
Wake-up time	92%
Get-out-of-bed time	100%
Subjective sleep quality	77%

NA (not applicable): Statement agreed from mixed e-Delphi findings, not assessed separately for level of agreement; LC (lack of consensus).

## Data Availability

Data generated within the study is provided in Supplementary Materials Table S3a–e.

## Supplementary Materials

The following supporting information can be downloaded at: https://www.mdpi.com/article/10.3390/cancers15153784/s1, References [45,46,47,48,49,50,51,52,53,54] are cited in the supplementary materials. Table S1. Potential rest-activity parameters. Table S2. Questions for round 1 of the e-Delphi study. Table S3a. Circadian rest-activity rhythms—group responses for individual statements and stability between rounds. Table S3b. Diagnostic criteria for circadian rest-activity rhythm disorders—group responses for individual statements and stability between rounds. Table S3c. A clinical assessment for cards—group responses for individual statements and stability between rounds. Table S3d. The use of actigraphy and other investigations—group responses for individual statements and stability between rounds. Table S3e. Additional statements—group responses for individual statements and stability between rounds.
