# Supplementary material for: International e-Delphi Consensus Recommendations for the Assessment and Diagnosis of Circadian rest–Activity Rhythm Disorders (CARDs) in Patients with Cancer
Source: Cancers (Basel). 2023 Jul 26;15(15):3784. doi: 10.3390/cancers15153784 (PMC10416864; doi:10.3390/cancers15153784)
Supplement: Supplementary file 1 [file cancers-15-03784-s001.zip › cancers-2464887-supplementary.pdf]

SUPPLEMENTARY TABLE S1. POTENTIAL REST-ACTIVITY PARAMETERS

**Accelerometry circadian measure**	**Description**
**General activity**	
Acrophase	The timing of peak physical activity (45)
Midline estimating statistic of rhythm (MESOR)	The average activity level over 24 hours (45)
Amplitude	A measure of half the extent of predictable variation within a cycle (46)
Relative amplitude	Empirical difference: (M10 – L5) / (M10 + L5) (47)
Intradaily variability (IV)	A measure of within-day rhythm fragmentation (47)
Interdaily stability (IS)	A measure of rhythm stability between days (47)
L5	Start timing of the 5 least active hours (47)
M10	Start timing of the 10 most active hours (47)
Circadian quotient	Amplitude / mesor (48)
Rhythm quotient	24 hour amplitude (A_{24HR}) / Relative amplitudes at 4, 8 and 12 hours (A_4+A_8+A_{12}) (48)
Dichotomy index (I<O)	Percentage of activity in bed (I) falling below the median activity out of bed(O) (45, 49)
Dichotomy index for daytime sedentariness	O<I percentage of activity out of bed which falls below median activity in bed (16)
Autocorrelation coefficient (r24)	Correlates activity at same time points between different days, considering consistency and regularity. Higher values are more stable (16, 49)
**Sleep-wake activity**	
Total time in bed	Time between into and out of bed time (15, 50)
Sleep onset latency	Number of minutes to fall asleep. Time between into bed time and sleep onset (15, 50, 51)
Wake after sleep onset	Sum of duration of all wake periods whilst in bed OR between sleep onset and offset (15, 52)
Total sleep time	Time between bedtime and wake time (52) OR total time in bed scored as asleep (15)
Wake minutes	Duration of wake during time in bed (15, 53)
Wake episodes / number of awakenings in the night (NWAK)	Number of wake episodes during sleep period (51)
Mean duration of wake episodes	Mean duration of all wake episodes (15)
Sleep fragmentation index (SFI)	Number of awakenings / total sleep time in minutes (15)
Sleep efficiency	Proportion of time asleep from time into bed until final time out of bed (15, 54)
Time napping and sleep minutes	Duration of sleep episodes during wake period (53)

SUPPLEMENTARY TABLE S2. QUESTIONS FOR ROUND 1 OF THE E-DELPHI STUDY.

**Part 1 – Rest-activity rhythms** – this section aims to establish if disordered rhythms occur in specific populations and if there are existing definitions or diagnostic criteria<br><br>1. Which serious diseases are associated with rest-activity circadian rhythm disorders? (Free text box)<br>2. Do rest-activity circadian rhythm disorders exist in patients with cancer? (Yes / No / Unsure)<br>3. If yes, are rest-activity circadian rhythm disorders in patients with cancer different from primary circadian sleep-wake rhythm disorders? (Yes / No / Unsure)<br>a. If you feel that they are different, please comment on how they are different (Free text box)<br>4. If yes, are rest-activity circadian rhythm disorders in patients with cancer different to those seen in other serious diseases (Yes / No / Unsure).<br>a. If you feel that they are different, please comment on how they are different (Free text box)<br>5. Are you aware of any published definitions of rest-activity circadian rhythm disorders? (Yes / No)<br>6. Are you aware of any published definitions of cancer-related rest-activity circadian rhythm disorders? (Yes / No)<br>7. If you are aware of any published definitions, please provide reference (Free text box)<br>8. Are you aware of any published diagnostic criteria for rest-activity rhythm disorders? (Yes / No)<br>9. Are you aware of any published diagnostic criteria for cancer-related rest-activity rhythm disorders? (Yes / No)<br>10. If you are aware of published diagnostic criteria, please provide reference (Free text box)<br>11. Do you have any other comments on existing definitions and/or criteria? (Free text box)
**Part 2 – Diagnostic criteria** – this section aims to establish diagnostic criteria<br><br>1. What do you think are the essential criteria for diagnosing a rest-activity circadian rhythm disorder? (Free text box)<br>2. What criteria are suggestive (not essential) for diagnosing a rest-activity circadian rhythm disorder? (Free text box)<br>3. What criteria are suggestive of a different cause of rest-activity rhythm disturbance? (Free text box)<br>4. Do you have any other comments about diagnostic criteria? (Free text box)
**Part 3 – Assessment of rest-activity rhythms** – this section aims to establish assessment methods (for diagnosis)<br><br>1. Is a clinical history needed to identify a rest-activity circadian rhythm disorder? (Yes / No – but it is supportive / No – it is not needed / Unsure)<br>2. If a clinical history is taken, what questions are important to identify a rest-activity circadian rhythm disorder? (Free text box)<br>3. Is a physical examination needed to identify a rest-activity circadian rhythm disorder? (Yes / No – but it is supportive / No – it is not needed / Unsure)<br>4. If a physical examination is performed, what physical signs are important to identify a rest-activity circadian rhythm disorder (Free text box)<br>5. Is actigraphy essential to identify a rest-activity circadian rhythm disorder? (Yes / No – but it is supportive / No – it is not needed / Unsure)<br>6. If actigraphy is used, what is the minimum period the patient should be monitored for? (days)<br>7. If actigraphy is used, what is the maximum period the patient should be monitored for? (days)<br>8. If actigraphy is used, what parameters should be measured, and what are the normal values for each parameter?<br>9. Is a sleep diary essential to identify a rest-activity rhythm disorder? (Yes / No – but it is supportive / No – it is not needed / Unsure)<br>10. If a sleep diary is used, how long should a patient be assessed?<br>11. If a sleep diary is used, what time points or events should be assessed?<br>12. Is an activity diary essential to identify a rest-activity rhythm disorder (Yes / No – but it is supportive / No – it is not needed / Unsure)

13. If an activity diary is used, how long should a patient be assessed?
14. If an activity diary is used, what time points or events should be assessed?
15. Are there any other investigations which are useful to identify a rest-activity circadian rhythm disorder? (Free text box)
16. Do you have any other comments about assessment? (Free text box)

SUPPLEMENTARY TABLE S3a. CIRCADIAN REST-ACTIVITY RHYTHMS - GROUP RESPONSES FOR INDIVIDUAL STATEMENTS AND STABILITY BETWEEN ROUNDS

Statement	Round 2					Round 3					Spearman's rank correlation coefficient R2 and R3	Sig.
	SD	D	U	A	SA	SD	D	U	A	SA		
Circadian rest-activity rhythm disorders ARE different from primary circadian sleep-wake disorders differ	0%	8%	31%	23%	38%	0%	0%	23%	15%	62%	0.647	**p=0.017**
	8%		31%	61%		0%		23%	**77%**			
The pathophysiology, clinical features, and consequences between circadian rest-activity and sleep-wake rhythm disorders differ	0%	15%	38%	23%	23%	0%	8%	31%	31%	31%	0.895	**p<0.001**
	15%		38%	46%		8%		31%	62%			
Circadian rest-activity rhythm considers the relationship between peak and trough activity levels	0%	15%	0%	62%	23%	0%	15%	0%	54%	31%	0.593	**p=0.033**
	15%		0%	**85%**		15%		0%	**85%**			
Circadian rest-activity rhythm considers physical activity across the 24-hour period	0%	0%	8%	38%	54%	0%	0%	8%	23%	69%	0.776	**p=0.002**
	0%		8%	**92%**		0%		8%	**92%**			
Circadian rest-activity rhythm considers the strength, or regularity, of rest and activity periods between days	0%	8%	8%	46%	38%	0%	8%	0%	38%	54%	0.442	p=0.130
	8%		8%	**84%**		8%		0%	**92%**			
Circadian rest-activity rhythm disorders do not focus on the timing of the sleep period	0%	54%	23%	15%	8%	0%	54%	23%	15%	8%	0.629	**p=0.021**
	54%		23%	23%		54%		23%	23%			
Patients with rest-activity rhythm disorders have dampened and disrupted circadian rhythms, rather than phase shifts or lengthening/shortening of the period	8%	0%	15%	69%	8%	8%	0%	15%	69%	8%	1.000	**p = 0.01**
	8%		15%	**77%**		8%		15%	**77%**			
Circadian rest-activity rhythm disorders are related to rest e.g. sleep fragmentation	8%	8%	0%	77%	8%	8%	8%	0%	77%	8%	1.000	**p = 0.01**
	16%		0%	**85%**		16%		0%	**85%**			
Circadian rest-activity disorders are related to activity e.g. reduced daytime activity levels	8%	8%	8%	46%	31%	8%	8%	8%	46%	31%	1.000	**p = 0.01**
	16%		8%	**77%**		16%		8%	**77%**			
	0%	15%	8%	46%	31%	0%	15%	0%	54%	31%	0.966	**p<0.001**

Circadian rest-activity rhythm disorders are related to reduced amplitude of activity levels (the difference between peak and trough levels)	15%		8%	**77%**		15%		0%	**85%**			
Circadian rest-activity rhythm disorders exist in patients with cancer and those receiving anticancer therapies	0%	0%	0%	54%	46%	0%	0%	0%	38%	62%	0.732	**p=0.004**
	0%		0%	**100%**		0%		0%	**100%**			
Circadian rest-activity rhythm disorders in patients with cancer ARE NOT different to circadian rest-activity rhythm disorders in patients with other serious diseases	0%	8%	62%	31%	0%	0%	8%	85%	8%	0%	0.639	**p=0.019**
	8%		62%	31%		8%		**85%**	8%			
There is not enough data to know if circadian rest-activity rhythm disorders are different between patients with cancer and other serious diseases	0%	23%	0%	54%	23%	0%	0%	23%	62%	15%	0.857	**P<0.001**
	23%		0%	**77%**		0%		23%	**77%**			
Circadian rest-activity rhythm disorders exist in patients with neurological disorders (e.g. Alzheimer's, Parkinson's, Delirium, Epilepsy, Traumatic brain injury)	0%	0%	0%	62%	38%	0%	0%	0%	54%	46%	0.854	**p<0.001**
	0%		0%	**100%**		0%		0%	**100%**			
Circadian rest-activity rhythm disorders exist in patients with sleep disorders (e.g. insomnia)	0%	8%	8%	77%	8%	0%	8%	8%	77%	8%	1.000	**p = 0.01**
	8%		8%	**85%**		8%		8%	**85%**			
Circadian rest-activity rhythm disorders exist in patients with respiratory disorders (e.g. obstructive sleep apnoea)	0%	8%	15%	69%	8%	0%	8%	15%	69%	8%	1.000	**p = 0.01**
	8%		15%	**77%**		8%		15%	**77%**			
Circadian rest-activity rhythm disorders exist in patients with psychiatric disorders (e.g. mood disorders, schizophrenia, eating disorders)	0%	0%	15%	77%	8%	0%	0%	0%	92%	8%	0.628	**p=0.022**
	0%		15%	**85%**		0%		0%	**100%**			
Circadian rest-activity rhythm disorders exist in patients with musculoskeletal disorders (e.g. osteoarthritis, inflammatory disorders)	0%	8%	38%	46%	8%	0%	8%	31%	54%	8%	0.910	**p<0.001**
	8%		38%	54%		8%		31%	62%			
Circadian rest-activity rhythm disorders exist in patients with acute illness (e.g. infection, hospitalisation)	0%	0%	8%	85%	8%	0%	0%	0%	85%	15%	0.544	p=0.055
	0%		8%	**93%**		0%		0%	**100%**			

Circadian rest-activity rhythm disorders exist in patients with ocular disorders (e.g. blindness)	0%	15%	38%	23%	23%	0%	15%	31%	31%	23%	0.958	**p<0.001**
	15%		38%	46%		15%		31%	54%			
Circadian rest-activity rhythm disorders exist in patients with endocrine disorders (e.g. diabetes, adrenal insufficiency)	0%	15%	38%	38%	8%	0%	15%	31%	38%	15%	0.916	**p<0.001**
	15%		38%	46%		15%		31%	53%			
Circadian rest-activity rhythm disorders exist in patients with gastrointestinal disorders (e.g. liver cirrhosis)	0%	8%	46%	46%	0%	0%	8%	38%	54%	0%	0.887	**p<0.001**
	8%		46%	46%		8%		38%	54%			
Circadian rest-activity rhythm disorders exist in patients with fibromyalgia and chronic fatigue syndrome	0%	8%	31%	54%	8%	0%	8%	31%	54%	8%	1.000	**p = 0.01**
	8%		31%	62%		8%		31%	62%			
Circadian rest-activity rhythm disorders exist in patients with cardiovascular disorders	0%	8%	31%	54%	8%	0%	8%	31%	46%	15%	0.955	**p<0.001**
	8%		31%	62%		8%		31%	61%			
Circadian rest-activity rhythm disorders exist in patients with fertility disorders	0%	8%	85%	8%	0%	0%	8%	85%	8%	0%	1.000	**p = 0.01**
	8%		**85%**	8%		8%		**85%**	8%			
Altered rest-activity rhythms can be a phenotype of other problems e.g. nocturia	0%	0%	23%	62%	15%	0%	0%	23%	62%	15%	1.000	**p = 0.01**
	0%		23%	**77%**		0%		23%	**77%**			
There are NO published diagnostic criteria for circadian rest-activity rhythm disorders, including cancer-related circadian rest-activity rhythm disorders	0%	8%	0%	54%	38%	0%	8%	0%	54%	38%	1.000	**p = 0.01**
	8%		0%	**92%**		8%		0%	**92%**			
Number of statements with consensus	**17/27 (63%)**					**19/27 (70%)**						

SD: Strongly disagree, D: Disagree, U: Unsure, A: Agree, SA: Strongly agree

SUPPLEMENTARY TABLE S3b. DIAGNOSTIC CRITERIA FOR CIRCADIAN REST-ACTIVITY RHYTHM DISORDERS - GROUP RESPONSES FOR INDIVIDUAL STATEMENTS AND STABILITY BETWEEN ROUNDS

**Statement**	**Round 2**					**Round 3**					**Spearman's rank correlation coefficient R2 and R3**	**Sig.**
	SD	D	U	A	SA	SD	D	U	A	SA		
**Essential clinical criteria**												
Evidence of a decreased daytime activity and increased night-time activity (e.g. daytime sedentariness and nocturnal restlessness)	0%	8%	0%	69%	23%	0%	8%	0%	69%	23%	1.000	**p = 0.01**
	8%		0%	**92%**		8%		0%	**92%**			
Evidence of fluctuating patterns of rest and activity between days	0%	15%	15%	54%	15%	0%	15%	8%	62%	15%	0.939	**p<0.001**
	15%		15%	**69%**		15%		8%	**77%**			
Evidence of physical activity spread across the 24-hour period, rather than focussed periods of rest and activity	0%	15%	15%	38%	31%	0%	0%	23%	46%	31%	0.960	**p<0.001**
	15%		15%	**69%**		0%		23%	**77%**			
Evidence of rest and activity periods that are not aligned with the 24-hour clock	0%	15%	8%	54%	23%	0%	15%	15%	54%	15%	0.737	**p=0.004**
	15%		8%	**77%**		15%		15%	**69%**			
The change in rest-activity rhythm occurred after the diagnosis of cancer or other serious disease	15%	23%	31%	23%	8%	15%	23%	31%	31%	0%	0.991	**p<0.001**
	38%		31%	31%		38%		31%	31%			
**Essential time duration criteria**												
The circadian rest-activity rhythm disorder should be present for 2 weeks or more	15%	31%	8%	31%	15%	15%	31%	0%	31%	23%	0.965	**p<0.001**
	46%		8%	46%		46%		0%	54%			
The circadian rest-activity rhythm disorder should be present for 1 month or more	15%	0%	31%	38%	15%	15%	0%	15%	62%	8%	0.834	**p<0.001**
	15%		31%	53%		15%		15%	**70%**			
**Essential impact criteria**												
The circadian rest-activity rhythm should have a clinical impact on the individual (i.e. fatigue, drowsiness, poor appetite)	0%	0%	15%	46%	38%	0%	0%	15%	46%	38%	1.000	**p = 0.01**
	0%		15%	**84%**		0%		15%	**84%**			
The circadian rest-activity rhythm should have an impact on family members or carer	23%	54%	15%	8%	0%	23%	62%	8%	8%	0%	0.939	**p<0.001**
	**77%**		15%	8%		**85%**		8%	8%			
**Essential criteria**												
There should be objective evidence of physical activity to diagnose a circadian rest-activity rhythm disorder (e.g. actigraphy)	0%	8%	0%	54%	38%	0%	8%	0%	54%	38%	1.000	**p = 0.01**
	8%		0%	**92%**		8%		0%	**92%**			

There should be objective evidence of biomarkers to diagnose a circadian rest-activity rhythm disorder (e.g. melatonin)	8%	46%	0%	46%	0%	8%	54%	0%	38%	0%	0.887	**p<0.001**
	52%		0%	46%		62%		0%	38%			
There should be subjective evidence of physical activity to diagnose a circadian rest-activity rhythm disorder (e.g. self-reported measures)	8%	23%	8%	54%	8%	8%	23%	0%	69%	0%	0.889	**p<0.001**
	31%		8%	62%		31%		0%	**69%**			
**Suggestive criteria**												
There should be subjective evidence of physical activity to diagnose a circadian rest-activity rhythm disorder (e.g. self-reported measures)	8%	15%	15%	54%	8%	8%	8%	15%	62%	8%	0.864	**p<0.001**
	23%		15%	62%		16%		15%	**70%**			
The individual has difficulty getting up and out of bed in the morning	8%	54%	15%	15%	8%	8%	62%	8%	23%	0%	0.778	**p=0.002**
	62%		15%	23%		**70%**		8%	23%			
The individual experiences fatigue that is not alleviated by rest	0%	38%	23%	31%	8%	0%	46%	8%	46%	0%	0.629	**p=0.021**
	38%		23%	39%		46%		8%	46%			
The individual naps in the day	0%	23%	31%	46%	0%	0%	15%	23%	62%	0%	0.712	**p=0.006**
	23%		31%	46%		15%		23%	62%			
The individual has disrupted sleep	0%	8%	15%	46%	31%	0%	0%	15%	54%	31%	0.908	**p<0.001**
	8%		15%	**77%**		0%		15%	**85%**			
The individual has low levels of physical activity	0%	23%	31%	23%	23%	0%	23%	38%	23%	15%	0.880	**p<0.001**
	23%		31%	46%		23%		38%	38%			
**Alternative causes of rest-activity rhythm disturbance**												
The use of sedatives	0%	8%	31%	54%	8%	0%	0%	31%	62%	8%	0.888	**p<0.001**
	8%		31%	62%		0%		31%	**70%**			
The use of stimulants	0%	8%	38%	46%	8%	0%	0%	38%	54%	8%	0.885	**p<0.001**
	8%		38%	54%		0%		38%	**62%**			
The use of steroids	0%	15%	54%	15%	15%	0%	15%	54%	15%	15%	0.867	**p<0.001**
	15%		54%	30%		15%		54%	30%			
The use of chemotherapy	8%	0%	8%	46%	38%	8%	0%	0%	62%	31%	0.852	**p<0.001**
	8%		8%	**84%**		8%		0%	**93%**			
The use of alcohol	0%	0%	31%	62%	8%	0%	0%	15%	77%	8%	0.753	**p=0.003**
	0%		31%	**70%**		0%		15%	**85%**			
Substance misuse / illicit drug use	0%	0%	54%	31%	15%	0%	0%	54%	38%	8%	0.974	**p<0.001**
	0%		54%	46%		0%		54%	46%			
Experiencing pain	0%	15%	8%	54%	23%	0%	15%	0%	62%	23%	0.953	**p<0.001**

	15%		8%	**77%**		15%		0%	**85%**			
Experiencing fatigue	0%	8%	31%	46%	15%	0%	8%	23%	54%	15%	0.928	**p<0.001**
	8%		31%	61%		8%		23%	**71%**			
Experiencing poor sleep	0%	0%	8%	77%	15%	0%	0%	8%	69%	23%	0.853	**p<0.001**
	0%		8%	**92%**		0%		8%	**92%**			
Experiencing urinary symptoms e.g. nocturia	0%	0%	31%	62%	8%	0%	0%	15%	77%	8%	0.753	**p=0.003**
	0%		31%	**70%**		0%		15%	**85%**			
Acute illness	8%	0%	15%	54%	23%	8%	0%	8%	62%	23%	0.939	**p<0.001**
	8%		15%	**77%**		8%		8%	**85%**			
Comorbid illness	0%	0%	15%	69%	15%	0%	0%	15%	69%	15%	1.000	**p = 0.01**
	0%		15%	**84%**		0%		15%	**84%**			
Diet	15%	8%	38%	38%	0%	15%	8%	38%	38%	0%	1.000	**p = 0.01**
	23%		38%	38%		23%		38%	38%			
Genetic evidence of short or long sleep times	8%	23%	46%	23%	0%	8%	23%	46%	23%	0%	1.000	**p = 0.01**
	31%		46%	23%		31%		46%	23%			
Hormonal imbalance	0%	8%	62%	15%	15%	0%	8%	62%	15%	15%	1.000	**p = 0.01**
	8%		62%	30%		8%		62%	30%			
Shift work	0%	0%	8%	38%	54%	0%	0%	0%	46%	54%	0.974	**p<0.001**
	0%		8%	**92%**		0%		0%	**100%**			
Jet lag	8%	8%	8%	54%	23%	8%	0%	0%	69%	23%	0.666	**p=0.013**
	16%		8%	**77%**		8%		0%	**92%**			
Number of statements with consensus	**17/35 (49%)**					**24/35 (69%)**						

SD: Strongly disagree, D: Disagree, U: Unsure, A: Agree, SA: Strongly agree

SUPPLEMENTARY TABLE S3c. A CLINICAL ASSESSEMENT FOR CARDS - GROUP RESPONSES FOR INDIVIDUAL STATEMENTS AND STABILITY BETWEEN ROUNDS

Statement	Round 2					Round 3					Spearman's rank correlation coefficient	Sig
	SD	D	U	A	SA	SD	D	U	A	SA		
A clinical history IS needed to identify a circadian rest-activity rhythm disorder and to consider alternative causes of a rest-activity rhythm disturbance	0%	0%	8%	38%	54%	0%	0%	8%	15%	77%	0.678	**p=0.011**
	0%		8%	**92%**		0%		8%	**92%**			
A clinical history should include an oncological history (cancer site, stage, site of metastases, and cancer treatments)	0%	0%	8%	46%	46%	0%	0%	8%	23%	69%	0.700	**p=0.008**
	0%		8%	**92%**		0%		8%	**92%**		1.000	
A clinical history should include medical, surgical and psychiatric comorbidities	0%	0%	0%	54%	46%	0%	0%	0%	46%	54%	0.857	**p<0.001**
	0%		0%	**100%**		0%		0%	**100%**			
A clinical history should include the use of medications (e.g. melatonin, beta blockers, steroids, stimulants, sedatives)	0%	0%	0%	46%	54%	0%	0%	0%	23%	77%	0.592	**p=0.033**
	0%		0%	**100%**		0%		0%	**100%**			
A clinical history should include daily routine and physical activity (e.g. duration and type of exercise)	0%	0%	0%	69%	31%	0%	0%	0%	62%	38%	0.843	**p<0.001**
	0%		0%	**100%**		0%		0%	**100%**			
A clinical history should include how long the individual has struggled with their rest / activity levels	0%	0%	0%	46%	54%	0%	0%	0%	31%	69%	0.720	**p=0.006**
	0%		0%	**100%**		0%		0%	**100%**			
A clinical history should include a sleep history	0%	0%	0%	38%	62%	0%	0%	0%	23%	77%	0.693	**p=0.009**
	0%		0%	**100%**		0%	0%	0%	**100%**			
A clinical history should include an assessment of their chronotype (own sleep time preference)	0%	0%	23%	38%	38%	0%	0%	8%	54%	38%	0.922	**p<0.001**
	0%		23%	**76%**		0%		8%	**92%**			
A clinical history should include an assessment of their greatest alertness (morning or evening person)	0%	15%	15%	46%	23%	0%	8%	8%	62%	23%	0.861	**p<0.001**
	15%		15%	**69%**		8%		8%	**85%**			
A clinical history should include sleep patterns of immediate family members (for genetic cause)	0%	31%	31%	38%	0%	0%	31%	15%	54%	0%	0.899	**p<0.001**
	31%		31%	38%		31%		15%	54%			
A clinical history should include assessing for symptoms (e.g. fatigue, daytime sleepiness, pain) and the timing of symptoms	0%	0%	0%	54%	46%	0%	0%	0%	46%	54%	0.857	**p<0.001**
	0%		0%	**100%**		0%		0%	**100%**			
A clinical history should include symptoms of hormonal imbalances or treatment for hormonal imbalances in family members	8%	23%	46%	15%	8%	8%	23%	46%	23%	0%	0.995	**p<0.001**
	31%		46%	23%		31%		46%	23%			
	0%	8%	0%	54%	38%	0%	0%	0%	54%	46%	0.835	**p<0.001**

A clinical history should include the individual's environment (e.g. family, newborns, occupation and shift work, long haul travel, light and noise exposure)	8%		0%	**92%**		0%		0%	**100%**			
A clinical history should include tobacco, alcohol, caffeine and illicit drug use	0%	8%	8%	62%	23%	0%	0%	0%	62%	38%	0.461	p=0.113
	8%		8%	**85%**		0%	0%	0%	**100%**			
A clinical history should include daytime sunlight / bright light exposure	0%	8%	8%	62%	23%	0%	8%	8%	62%	23%	1.000	**p = 0.01**
	8%		8%	**85%**		8%		8%	**85%**			
A clinical history should include health behaviour history	0%	0%	31%	46%	23%	0%	0%	23%	46%	31%	0.878	**p<0.001**
	0%		31%	**69%**		0%		23%	**77%**			
A physical examination is NOT needed to identify a circadian rest-activity rhythm disorder	23%	23%	23%	23%	8%	23%	23%	23%	31%	0%	0.991	**p<0.001**
	46%		23%	31%		46%		23%	31%			
A physical examination should consider a musculoskeletal examination	0%	23%	38%	38%	0%	0%	23%	38%	38%	0%	1.000	**p = 0.01**
	23%		38%	38%		23%		38%	38%			
A physical examination should include an eye examination (e.g. visual impairment)	0%	31%	38%	31%	0%	0%	31%	38%	31%	0%	1.000	**p = 0.01**
	31%		38%	31%		31%		38%	31%			
A physical examination should include assessing body mass index	8%	23%	23%	46%	0%	8%	23%	8%	62%	0%	0.897	**p<0.001**
	31%		23%	46%		31%		8%	62%			
Number of statements with consensus	**14/20 (70%)**					**14/20 (70%)**						

SD: Strongly disagree, D: Disagree, U: Unsure, A: Agree, SA: Strongly agree

SUPPLEMENTARY TABLE 3d. THE USE OF ACTIGRAPHY AND OTHER INVESTIGATIONS - GROUP RESPONSES FOR INDIVIDUAL STATEMENTS AND STABILITY BETWEEN ROUNDS

**Statement**	**Round 2**					**Round 3**					**Spearman's rank correlation coefficient**	**Sig.**
	SD	D	U	A	SA	SD	D	U	A	SA		
Actigraphy is ESSENTIAL to identify a circadian rest-activity rhythm disorder	8%	0%	8%	23%	62%	8%	0%	0%	15%	77%	0.796	**p=0.001**
	8%		8%	**85%**		8%		0%	**92%**			
Actigraphy is SUPPORTIVE in identifying a circadian rest-activity rhythm disorder	8%	31%	0%	15%	46%	8%	31%	0%	8%	54%	0.955	**p<0.001**
	39%		0%	61%		39%		0%	62%			
The MINIMUM period actigraphy should be used for is 3 days	8%	38%	8%	23%	23%	8%	46%	8%	23%	15%	0.812	**p<0.001**
	46%		8%	46%		54%		8%	38%			
The MINIMUM period actigraphy should be used for is 5 days	8%	69%	8%	8%	8%	8%	69%	8%	8%	8%	1.000	**p = 0.01**
	**77%**		8%	16%		**77%**		8%	16%			
The MINIMUM period actigraphy should be used for is 14 days	15%	38%	15%	31%	0%	15%	38%	15%	31%	0%	1.000	**p = 0.01**
	53%		15%	31%		53%		15%	31%			
The MAXIMUM period actigraphy should be used for is 7 days	15%	38%	15%	15%	15%	15%	38%	15%	15%	15%	1.000	**p = 0.01**
	53%		15%	30%		53%		15%	30%			
The MAXIMUM period actigraphy should be used for is 14 days	8%	38%	8%	38%	8%	8%	38%	8%	46%	0%	0.977	**p<0.001**
	46%		8%	46%		46%		8%	46%			
The MAXIMUM period actigraphy should be used for is 30 days	23%	62%	15%	0%	0%	23%	62%	15%	0%	0%	1.000	**p = 0.01**
	**85%**		15%	0%		**85%**		15%	0%			
The MAXIMUM period actigraphy should be used for is 56 days	31%	54%	8%	8%	0%	31%	62%	8%	0%	0%	0.920	**p<0.001**
	**85%**		8%	8%		**93%**		8%	0%			
There is no maximum period actigraphy should be used	15%	38%	15%	23%	8%	15%	31%	15%	31%	8%	0.900	**p<0.001**
	53%		15%	31%		46%		15%	39%			
When using actigraphy, the watch should be worn on the DOMINANT wrist	23%	62%	0%	0%	15%	23%	69%	0%	0%	8%	0.915	**p<0.001**
	**85%**		0%	15%		**92%**		0%	8%			
When using actigraphy, the watch should be worn on the NON-DOMINANT wrist	0%	8%	0%	62%	31%	0%	0%	0%	69%	31%	0.931	**p<0.001**
	8%		0%	**93%**		0%		0%	**100%**			
**Important and clinically relevant rest-activity parameter**												
Physical activity mesor (rhythm adjusted average activity level of the day)	15%	8%	0%	69%	8%	15%	8%	0%	69%	8%	1.000	**p = 0.01**
	23%		0%	**77%**		23%		0%	**77%**			
	0%	8%	8%	54%	31%	0%	8%	8%	54%	31%	1.000	**p = 0.01**

Physical activity amplitude (the different between peak and trough activity levels)	8%		8%	**85%**		8%		8%	**85%**			
Physical activity acrophase (timing of peak activity)	8%	8%	15%	46%	23%	8%	0%	23%	54%	15%	0.619	**p=0.024**
	16%		15%	**69%**		8%		23%	**69%**			
Peak activity level	8%	23%	8%	46%	15%	8%	8%	8%	69%	8%	0.736	**p=0.004**
	31%		8%	61%		16%		8%	**77%**			
Physical activity period (time period of physical activity)	0%	8%	0%	85%	8%	0%	8%	0%	92%	0%	0.736	**p=0.004**
	8%		0%	**93%**		8%		0%	**92%**			
Interdaily stability (a measure of rhythm stability between days)	0%	0%	0%	69%	31%	0%	0%	0%	77%	23%	0.822	**p<0.001**
	0%		0%	**100%**		0%		0%	**100%**			
Intra-daily variability (a measure of rhythm fragmentation)	0%	0%	15%	69%	15%	0%	0%	8%	77%	15%	0.879	**p<0.001**
	0%		15%	**84%**		0%		8%	**92%**			
Dichotomy index (percentage of in bed activity counts less than median out of bed activity counts)	0%	0%	15%	31%	54%	0%	0%	15%	31%	54%	1.000	**p = 0.01**
	0%		15%	**85%**		0%		15%	**85%**			
Daytime sedentariness (percentage of activity out of bed which falls below the median activity in bed)	8%	0%	15%	38%	38%	8%	0%	0%	54%	38%	0.944	**p<0.001**
	8%		15%	**76%**		8%		0%	**92%**			
24-hour autocorrelation coefficient (r24) (a measure of assessing regularity of activity between 24 hour periods)	0%	8%	15%	38%	38%	0%	8%	15%	31%	46%	0.930	**p<0.001**
	8%		15%	**76%**		8%		15%	**77%**			
M10 (mean timing of 10 most active hours)	8%	23%	8%	54%	8%	8%	23%	0%	62%	8%	0.953	**p<0.001**
	31%		8%	62%		31%		0%	**70%**			
L5 (mean timing of 5 least active hours)	8%	23%	15%	54%	0%	8%	31%	0%	62%	0%	0.923	**p<0.001**
	31%		15%	54%		39%		0%	62%			
Rhythm quotient (a measure of activity and sleep consolidation, considering amplitudes at 4-, 8-, 12-, and 24-hour periods)	0%	15%	31%	54%	0%	0%	15%	31%	54%	0%	1.000	**p = 0.01**
	15%		31%	54%		15%		31%	54%			
Circadian quotient (a measure of circadian rhythm strength, ratio of amplitude to mesor)	0%	15%	23%	38%	23%	0%	15%	23%	46%	15%	0.958	**p<0.001**
	15%		23%	61%		15%		23%	61%			
Time spent in bed (time between getting in and out of bed)	0%	15%	0%	69%	15%	0%	15%	0%	69%	15%	1.000	**p = 0.01**
	15%			**84%**		15%		0%	**84%**			
Sleep onset (beginning of sleep period)	8%	8%	15%	46%	23%	8%	8%	8%	54%	23%	0.956	**p<0.001**
	16%		15%	**69%**		16%		8%	**77%**			
Sleep offset (end of sleep period)	8%	8%	15%	46%	23%	8%	8%	8%	54%	23%	0.956	**p<0.001**
	16%		15%	**69%**		16%		8%	**77%**			
	0%	15%	8%	62%	15%	0%	15%	0%	69%	15%	0.933	**p<0.001**

Total sleep time (total time asleep between in and out of bed time)	15%		8%	**77%**		15%		0%	**84%**			
Sleep duration (time between sleep onset and offset)	0%	23%	8%	62%	8%	0%	15%	0%	77%	8%	0.796	**p=0.001**
	23%		8%	**70%**		15%		0%	**85%**			
Sleep efficiency (percentage of time asleep between sleep onset and offset)	0%	15%	23%	38%	23%	0%	8%	15%	46%	31%	0.823	**p<0.001**
	15%		23%	61%		8%		15%	**77%**			
Sleep fragmentation index (number of awakenings to total sleep time)	0%	15%	15%	31%	38%	0%	15%	0%	38%	46%	0.912	**p<0.001**
	15%		15%	**69%**		15%		0%	**84%**			
Wake after sleep onset (number of minutes awake between sleep onset and offset)	0%	23%	23%	38%	15%	0%	23%	15%	38%	23%	0.931	**p<0.001**
	23%		23%	53%		23%		15%	61%			
Sleep onset latency (the number of minutes from getting into bed and falling to sleep)	8%	23%	15%	38%	15%	8%	23%	8%	46%	15%	0.968	**p<0.001**
	31%		15%	53%		31%		8%	61%			
Number of night-time awakenings	0%	15%	8%	54%	23%	0%	15%	8%	54%	23%	1.000	**p = 0.01**
	15%		8%	**77%**		15%		8%	**77%**			
Duration of night-time awakenings	0%	15%	8%	62%	15%	0%	15%	8%	62%	15%	1.000	**p = 0.01**
	15%		8%	**77%**		15%		8%	**77%**			
Number of daytime naps	0%	15%	0%	62%	23%	0%	15%	0%	62%	23%	1.000	**p = 0.01**
	15%		0%	**85%**		15%		0%	**85%**			
Duration of daytime naps	8%	0%	8%	54%	31%	8%	0%	8%	46%	38%	0.910	**p<0.001**
	8%		8%	**85%**		8%		8%	**84%**			
**Patient diary**												
A patient diary is ESSENTIAL to identifying a circadian rest-activity disorder	8%	31%	23%	8%	31%	8%	31%	38%	0%	23%	0.949	**p<0.001**
	39%		23%	39%		39%		38%	23%			
A patient diary is SUPPORTIVE in identifying a circadian rest-activity rhythm disorder	0%	15%	0%	77%	8%	0%	15%	0%	77%	8%	1.000	**p = 0.01**
	15%		0%	**79%**		15%		0%	**85%**			
A patient diary supports the use of actigraphy in identifying a circadian rest-activity rhythm disorder	8%	0%	0%	62%	31%	8%	0%	0%	62%	31%	1.000	**p = 0.01**
	8%		0%	**93%**		8%		0%	**93%**			
Without actigraphy, a patient diary is not necessary to identify a circadian rest-activity rhythm disorder	8%	31%	38%	15%	8%	8%	38%	31%	15%	8%	0.946	**p<0.001**
	39%		38%	23%		46%		31%	23%			
To help identify a circadian rest-activity rhythm disorder, a patient diary should be used for 5 days	0%	62%	23%	15%	0%	0%	69%	15%	15%	0%	0.895	**p<0.001**
	62%		23%	15%		**69%**		15%	15%			
To help identify a circadian rest-activity rhythm disorder, a patient diary should be used for 7 days	0%	38%	15%	31%	15%	0%	38%	15%	31%	15%	0.946	**p<0.001**
	38%		15%	46%		38%		15%	46%			
To help identify a circadian rest-activity rhythm disorder, a patient diary should be used for 14 days	8%	31%	8%	46%	8%	8%	31%	8%	46%	8%	1.000	**p = 0.01**
	39%		8%	54%		39%		8%	54%			

To help identify a circadian rest-activity rhythm disorder, a patient diary should be used for the duration of actigraphy monitoring	0%	0%	0%	46%	54%	0%	0%	0%	31%	69%	0.720	**p=0.006**
	0%		0%	**100%**		0%	0%	0%	**100%**			
To help identify a circadian rest-activity rhythm disorder, a patient diary should include the patient's bedtime	8%	0%	8%	54%	31%	8%	0%	8%	46%	38%	0.910	**p<0.001**
	8%		8%	**85%**		8%		8%	**84%**			
To help identify a circadian rest-activity rhythm disorder, a patient diary should include the patient's time to lights out	8%	8%	0%	54%	31%	8%	8%	0%	46%	38%	0.910	**p<0.001**
	16%		0%	**85%**		16%		0%	**84%**			
To help identify a circadian rest-activity rhythm disorder, a patient diary should include the patient's sleep onset time	8%	15%	8%	38%	31%	8%	8%	8%	38%	38%	0.895	**p<0.001**
	23%		8%	**69%**		16%		8%	**76%**			
To help identify a circadian rest-activity rhythm disorder, a patient diary should include the patient's sleep latency (time from getting into bed and falling asleep)	8%	15%	8%	38%	31%	8%	8%	8%	38%	38%	0.895	**p<0.001**
	23%		8%	**69%**		16%		8%	**76%**			
To help identify a circadian rest-activity rhythm disorder, a patient diary should include the patient's time and duration of awakenings from sleep	8%	15%	0%	54%	23%	8%	8%	0%	62%	23%	0.939	**p<0.001**
	23%		0%	**77%**		16%		0%	**85%**			
To help identify a circadian rest-activity rhythm disorder, a patient diary should include the patient's wake up time	0%	8%	0%	54%	38%	0%	8%	0%	54%	38%	1.000	**p = 0.01**
	8%		0%	**92%**		8%		0%	**92%**			
To help identify a circadian rest-activity rhythm disorder, a patient diary should include the patient's get out of bed time	0%	0%	0%	62%	38%	0%	0%	0%	62%	38%	1.000	**p = 0.01**
	0%		0%	**100%**		0%		0%	**100%**			
To help identify a circadian rest-activity rhythm disorder, a patient diary should include the patient's pain level at bed and wake time	8%	15%	23%	31%	23%	8%	15%	23%	31%	23%	1.000	**p = 0.01**
	23%		23%	54%		23%		23%	54%			
To help identify a circadian rest-activity rhythm disorder, a patient diary should include the patient's fatigue level at bed and wake time	8%	8%	23%	46%	15%	8%	8%	23%	46%	15%	1.000	**p = 0.01**
	16%		23%	61%		16%		23%	61%			
To help identify a circadian rest-activity rhythm disorder, a patient diary should include the patient's nap time and duration	8%	0%	8%	54%	31%	8%	0%	0%	62%	31%	0.952	**p<0.001**
	8%		8%	**85%**		8%		0%	**93%**			
To help identify a circadian rest-activity rhythm disorder, a patient diary should include the patient's level of daytime sleepiness	8%	8%	0%	62%	23%	8%	8%	0%	62%	23%	1.000	**p = 0.01**
	16%		0%	**85%**		16%		0%	**85%**			
To help identify a circadian rest-activity rhythm disorder, a patient diary should include the patient's time of meals	0%	31%	38%	23%	8%	0%	38%	31%	31%	0%	0.728	**p=0.005**
	31%		38%	31%		38%		31%	31%			
To help identify a circadian rest-activity rhythm disorder, a patient diary should include the patient's time of physical activity	0%	8%	15%	69%	8%	0%	0%	8%	77%	15%	0.609	**p=0.027**
	8%		15%	**77%**		0%		8%	**92%**			

To help identify a circadian rest-activity rhythm disorder, a patient diary should include the patient's description and duration of physical activity	0%	8%	15%	69%	8%	0%	8%	8%	69%	15%	0.798	**p=0.001**
	8%		15%	**77%**		8%		8%	**84%**			
To help identify a circadian rest-activity rhythm disorder, a patient diary should include the patient's perceived level of exertion during activity	8%	23%	15%	46%	8%	8%	23%	0%	62%	8%	0.923	**p<0.001**
	31%		15%	54%		31%		0%	**70%**			
To help identify a circadian rest-activity rhythm disorder, a patient diary should include the patient's symptoms during activity (e.g. fatigue or pain)	8%	23%	23%	38%	8%	8%	15%	8%	69%	0%	0.735	**p=0.004**
	31%		23%	46%		23%		8%	**69%**			
To help identify a circadian rest-activity rhythm disorder, a patient diary should include the times the device was removed or replaced	8%	0%	0%	62%	31%	8%	0%	0%	62%	31%	1.000	**p = 0.01**
	8%		0%	**93%**		8%		0%	**93%**			
To help identify a circadian rest-activity rhythm disorder, a patient diary should include the patient's time spent in a dark room resting e.g. watching a movie	8%	23%	31%	23%	15%	8%	23%	31%	31%	8%	0.985	**p<0.001**
	31%		31%	38%		31%		31%	39%			
To help identify a circadian rest-activity rhythm disorder, a patient diary should include the patient's time spent indoors and outdoors	8%	31%	15%	38%	8%	8%	31%	8%	46%	8%	0.968	**p<0.001**
	39%		15%	46%		39%		8%	54%			
To help identify a circadian rest-activity rhythm disorder, a patient diary should include the patient's mood	8%	31%	31%	15%	15%	8%	31%	31%	15%	15%	1.000	**p = 0.01**
	39%		31%	30%		39%		31%	30%			
To help identify a circadian rest-activity rhythm disorder, a patient diary should include the patient's acute stressful events	8%	23%	38%	23%	8%	8%	23%	38%	23%	8%	1.000	**p = 0.01**
	31%		38%	31%		31%		38%	31%			
To help identify a circadian rest-activity rhythm disorder, a patient diary should include the patient's subjective sleep quality	8%	0%	15%	54%	23%	8%	0%	15%	54%	23%	1.000	**p = 0.01**
	8%		15%	**77%**		8%		15%	**77%**			
To help identify a circadian rest-activity rhythm disorder, a patient diary should include the patient's pre-bedtime activities	8%	31%	23%	31%	8%	8%	31%	8%	46%	8%	0.947	**p<0.001**
	39%		23%	39%		39%		8%	54%			
To help identify a circadian rest-activity rhythm disorder, a patient diary should include the patient's use of alcohol, caffeine, and smoking	0%	8%	0%	85%	8%	0%	15%	0%	77%	8%	0.817	**p<0.001**
	8%		0%	**93%**		15%		0%	**85%**			
To help identify a circadian rest-activity rhythm disorder, a patient diary should include the patient's use of medications	0%	15%	0%	69%	15%	0%	15%	0%	69%	15%	1.000	**p = 0.01**
	15%		0%	**84%**		15%		0%	**84%**			

To help identify a circadian rest-activity rhythm disorder, a patient diary should include the patient's most active time period	8%	31%	31%	23%	8%	8%	38%	31%	15%	8%	0.838	**p<0.001**
	39%		31%	31%		46%		31%	13%			
To help identify a circadian rest-activity rhythm disorder, a patient diary should include the patient's most restful time period	8%	31%	31%	23%	8%	8%	38%	31%	15%	8%	0.838	**p<0.001**
	39%		31%	31%		46%		31%	13%			
Additional investigations to identify a circadian rest-activity rhythm disorder include the circadian melatonin measures	8%	23%	8%	62%	0%	8%	23%	8%	62%	0%	1.000	**p = 0.01**
	31%		8%	62%		31%		8%	62%			
Additional investigations to identify a circadian rest-activity rhythm disorder include blood tests (full blood count, thyroid function, metabolic panel)	8%	31%	31%	23%	8%	8%	31%	31%	23%	8%	1.000	**p = 0.01**
	39%		31%	31%		39%		31%	31%			
Additional investigations to identify a circadian rest-activity rhythm disorder include circadian body temperature	8%	23%	15%	38%	15%	8%	23%	15%	46%	8%	0.968	**p<0.001**
	31%		15%	53%		31%		15%	52%			
Additional investigations to identify a circadian rest-activity rhythm disorder include measures of bedroom environment (e.g. light, temperature, sound)	8%	23%	23%	38%	8%	8%	23%	8%	54%	8%	0.926	**p<0.001**
	31%		23%	46%		31%		8%	64%			
Additional investigations to identify a circadian rest-activity rhythm disorder include measures of daytime environment (e.g. light, temperature, sound)	8%	23%	31%	31%	8%	8%	23%	31%	31%	8%	1.000	**p = 0.01**
	31%		31%	39%		31%		31%	39%			
Number of statements with consensus	**43/79 (54%)**					**49/79 (62%)**						

SD: Strongly disagree, D: Disagree, U: Unsure, A: Agree, SA: Strongly agree

SUPPLEMENTARY TABLE 3e. ADDITIONAL STATEMENTS - GROUP RESPONSES FOR INDIVIDUAL STATEMENTS AND STABILITY BETWEEN ROUNDS

Round 3 modified statement				SD	D	U	A	SA
When using actigraphy, the watch should be worn on the non-dominant wrist unless there is a medical reason not to do so				0%	0%	0%	31%	69%
				0%		0%	**100%**	

Round 3 new statements				SD	D	U	A	SA
Devices that measure rest and activity, including accelerometry, are essential to identifying a circadian rest-activity rhythm disorder				15%	15%	15%	46%	8%
				30%		15%	54%	
Rest-activity rhythms can be assess using accelerometry at multiple sites, including wrist, chest, hip or leg				0%	0%	0%	62%	38%
				0%	0%	0%	**100%**	

SD: Strongly disagree, D: Disagree, U: Unsure, A: Agree, SA: Strongly agre
